# Clinical Profiles and Medication Predictors in Early Childhood Psychiatric Referrals: A 10-Year Retrospective Study

**DOI:** 10.3390/medicina61061038

**Published:** 2025-06-04

**Authors:** Leyla Bozatlı, Hasan Cem Aykutlu, Cansu Uğurtay Dayan, Tuğçe Ataş, Esra Nisa Arslan, Yeşim Özge Gündüz Gül, Işık Görker

**Affiliations:** 1 Department of Child and Adolescent Psychiatry, Trakya University Faculty of Medicine, Edirne 22000, Türkiye; hasancemaykutlu@trakya.edu.tr (H.C.A.); isikgorker@trakya.edu.tr (I.G.); 2 Department of Child and Adolescent Psychiatry, Kartal Dr. Lütfi Kırdar City Hospital, İstanbul 34862, Türkiye; cansuugurtay@gmail.com; 3 Department of Child and Adolescent Psychiatry, Artvin State Hospital, Artvin 08000, Türkiye; tugce.atas@saglik.gov.tr; 4 Department of Child and Adolescent Psychiatry, Patnos State Hospital, Ağrı 04500, Türkiye; nisayduk@gmail.com; 5 Department of Physical Medicine and Rehabilitation, Ankara Pursaklar State Hospital, Ankara 06145, Türkiye; ozgeyesimgunduz@gmail.com

**Keywords:** preschool mental health, preschooler, early diagnosis, psychiatric disorders, psychotropic medication predictors

## Abstract

*Background and Objectives*: Although psychiatric disorders in early childhood are increasingly recognized, comprehensive clinical data from large samples in this age group remain limited. This study presents one of the largest and longest-term evaluations in Türkiye of children aged 0–72 months referred to child psychiatry. It aims to identify the most common presenting complaints, diagnostic patterns, and key predictors of psychotropic medication initiation in a previously understudied age group. *Materials and Methods*: This retrospective analysis included 3312 children aged 0–72 months who presented to the outpatient child psychiatry clinic of Trakya University Medical Faculty Hospital in Edirne, Türkiye. Clinical records were reviewed to extract data on presenting complaints, psychiatric diagnoses, psychotropic medication initiation, and demographic details, including age and sex. *Results*: The most common presenting complaints were “delayed speech development”, “irritability/frustration”, “hyperactivity”, “requests for medical reports”, and “stuttering.” These complaints were more prevalent among children who received psychiatric diagnoses. Psychiatric diagnoses were more common in boys. Boys also presented at older ages and had longer follow-up durations. Psychotropic medications were initiated in 26.9% of the cases. The most frequently reported side effects were loss of appetite and drowsiness. Logistic regression analysis revealed that specific complaints were significantly predictive of initiating medication. These included “inability to speak”, “irritability/frustration”, “hyperactivity”, “lack of eye contact”, “aggression”, “school refusal”, “sleep problems”, and “fears.” *Conclusions*: This study revealed that some early childhood complaints, such as “inability to speak”, “restlessness”, “hyperactivity”, and “not making eye contact”, are strong predictors of both psychiatric diagnosis and initiation of psychotropic medication. The findings highlight the usefulness of structured assessment protocols in early childhood psychiatric services. The implementation of systematic screening for risk symptoms may facilitate early diagnosis and support more appropriate and timely treatment approaches, especially in resource-limited regions.

## 1. Introduction

The first six years of a child’s life constitute a critical period during which the fundamental building blocks of development are established. This period is characterized by significant transformations in physical, mental, emotional, and social domains, along with rapid brain growth. Critical developmental milestones, including language acquisition, motor skill refinement, and the formation of emotional bonds, occur prominently during this time. Early childhood, particularly between the ages of 0 and 6, is highly critical for an individual’s emotional, cognitive, and social development. Early experiences and environmental factors can have a lasting impact on this process. Each step in mental and emotional development during this phase can have a direct influence on the child’s future life [1]. Therefore, accurately assessing preschool children’s behaviors, supporting their developmental progress, and timely identification and intervention for potential psychological or emotional issues are essential. Early diagnosis and appropriate interventions can help prevent psychological difficulties in later years and facilitate the acquisition of healthy emotional and social competencies. Consequently, timely and proper assessment and treatment of childhood and adolescent psychopathology are emphasized as critical measures to mitigate future difficulties. Providing effective support during early childhood is, thus, vital for promoting psychological health, social adjustment, and overall well-being [2].

While it is recognized that children under 7 years old may display mental health symptoms with potentially lasting effects on their development and overall well-being [3], research into the prevalence of mental disorders at this young age remains limited [4]. Gaining a clearer understanding of the prevalence and comorbidity of mental disorders in early childhood is crucial for effective service planning, treatment interventions, and the development of appropriate assessment tools. Nevertheless, several challenges complicate the classification of mental disorders in young children, primarily due to the profound developmental changes occurring during the first seven years of life. Because early childhood is characterized by rapid physical, emotional, behavioral, and cognitive growth, emotional and behavioral difficulties are often perceived as temporary and, thus, are not typically classified as mental disorders [5,6]. Additionally, distinguishing between normal developmental behaviors and psychopathological conditions can be inherently difficult. For example, oppositional behavior, which tends to increase between ages two and four, is considered a vital developmental phase for achieving autonomy and is only classified as a mental disorder if significant functional impairment occurs. Furthermore, because interactions with their caregivers closely influence young children’s emotional and behavioral responses, it can be challenging to determine whether these issues indicate individual psychopathology or are the result of a dysfunctional caregiver–child relationship [7].

Epidemiological studies indicate that mental disorders affect approximately 16–18% of school-age children and adolescents, with a high risk of these problems persisting into later adolescence and adulthood [8]. While the importance of early symptom recognition and referral for treatment is widely emphasized in child psychiatry, epidemiological research focusing on preschool-aged children remains limited, with even fewer studies targeting children younger than four years old [9]. One of the few studies examining outpatient clinic admissions for children aged 0–5 years reported that 71.6% of these children received a psychiatric diagnosis [10]. Another study assessing psychiatric symptoms in three-year-old children based on parental reports identified behavioral problems in 35.6% of the children evaluated [11], while research on children aged 1–2 years reported that 10–15% exhibited social–emotional or behavioral issues [12]. A meta-analysis incorporating 10 epidemiological studies from eight countries, including data from 18,282 children aged between 12 and 83 months, found an overall prevalence rate of mental disorders of 20.1%. Among these, anxiety disorders (8.5%), oppositional defiant disorder (4.9%), and Attention Deficit Hyperactivity Disorder (4.3%) were the most frequently observed conditions. Moreover, psychiatric comorbidity was observed in 6.4% of the cases [6]. Many mental disorders identified during the preschool years can be effectively managed through psychosocial interventions, which are considered first-line treatments. Nevertheless, psychotropic medications may be necessary if mental health symptoms persist despite adequate psychosocial support [13,14].

The use of psychotropic medications during the preschool years to manage mental, behavioral, and emotional disorders—conditions that profoundly affect the well-being of both children and their families—has shown a steady increase in recent years [15,16,17]. Reported initiation rates of pharmacological treatment in children under six years presenting to child psychiatry clinics vary widely, ranging from 6.8% [18] to 7.3% [19] and up to 24.2% [20]. Despite this trend, significant gaps remain in the literature regarding clinical decision-making processes and the broader context of psychotropic medication use in early childhood. This study aims to address these gaps by providing a comprehensive overview of clinical presentation patterns, diagnostic distribution, and treatment initiation practices in children aged 0–6 years admitted to outpatient psychiatric services. In particular, we investigate the prevalence of specific psychiatric diagnoses, the frequency and determinants of psychotropic drug initiation, commonly prescribed medications, and their observed adverse effects, an area that remains underexplored in this age group. By presenting empirical evidence, our findings aim to support clinicians in making informed safe prescribing decisions and to assist researchers in identifying high-risk symptom profiles and diagnostic markers that can inform targeted interventions and enhance mental health service planning.

## 2. Materials and Methods

Medical records of patients younger than six years admitted to the Child and Adolescent Mental Health and Diseases Outpatient Clinic at Trakya University Medical School Hospital from 1 January 2007 to 31 December 2016 were retrospectively analyzed. The hospital’s electronic records system identified 4293 cases admitted during this 10-year period. After excluding cases aged 73 months or older and those with incomplete data, 3312 cases remained eligible for analysis.

Data collected included age, sex, date of admission, complaints reported by family members, medication usage, medication-related adverse effects, psychiatric diagnoses, parental education levels, the presence of psychiatric illnesses, and psychiatric medication treatment history.

Psychiatric diagnoses were determined according to the Diagnostic and Statistical Manual of Mental Disorders, Fourth/Fifth Editions (DSM IV-TR/DSM-5). Children who showed symptoms due to adverse psychosocial conditions or difficulties in the parent–child relationship but did not meet the DSM criteria for Reactive Attachment Disorder (RAD) were diagnosed with “stimulus deprivation.” Both conditions were categorized under the broader concept of attachment disorders. The Denver II Developmental Screening Test was also administered to participants diagnosed with general developmental delay (GDD).

### 2.1. Ethics Approval

This retrospective study used medical records from the hospital’s database. The data were accessed for research between 15 July 2018 and 15 July 2020. During the data collection phase, the authors accessed identifiable information necessary for accurate data extraction (e.g., to avoid duplication or confirm inclusion criteria). However, all personal identifiers were removed, and the data were anonymized before analysis to ensure participant confidentiality. The study protocol was approved by the Trakya University Faculty of Medicine Dean’s Office Scientific Research Ethics Committee (2018/46), and all procedures complied with relevant data protection regulations.

### 2.2. Statistical Analysis

All statistical analyses were performed using SPSS version 22.0 (IBM SPSS Statistics for Windows, Version 20.0. Armonk, NY: IBM Corp.). Descriptive statistics were used to summarize the study data; continuous variables were expressed as mean ± standard deviation (SD), and categorical variables were presented as frequencies and percentages. The Kolmogorov–Smirnov test was employed to assess the normality of continuous variables. Continuous variables that did not demonstrate normal distribution were compared between the two groups using the Mann–Whitney U test. Differences between categorical variables were evaluated using the Chi-Square (χ^2^) test; Bonferroni correction was applied to adjust for multiple testing when multiple category comparisons were made. Logistic regression analysis was performed to investigate the effects of independent variables on the dependent variables, and the results were expressed as odds ratios (OR) with 95% confidence intervals (CI). A *p*-value of less than 0.05 was considered statistically significant for all analyses.

## 3. Results

Of the 3312 cases included in the study, 1174 (35.4%) were girls and 2138 (64.6%) were boys. The patients ranged in age from 1 to 72 months, with a mean age of 46.59 ± 16.48 months. The educational status of the patients, parental education levels, and parental psychiatric illness status are summarized in Table 1.

**Figure 1:**
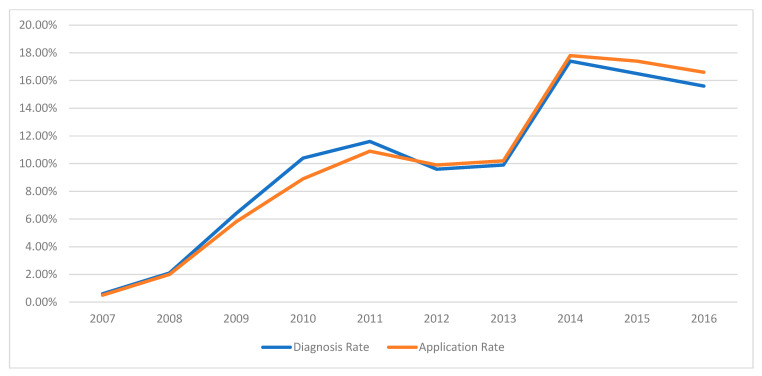
Distribution of the number of applications and the proportion of children diagnosed by year.

**Figure 2:**
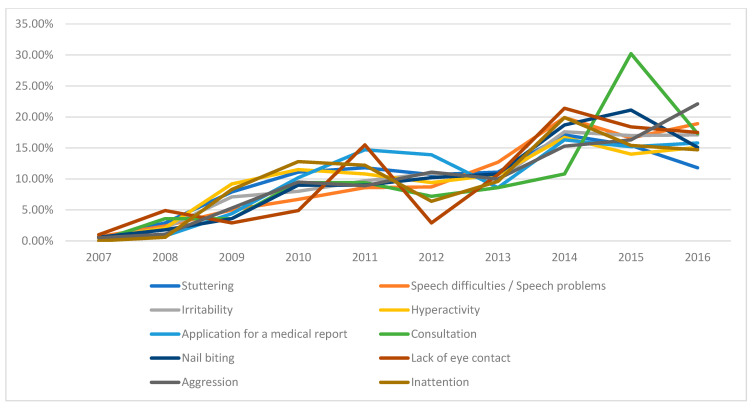
Distribution of application complaints by year.

**Figure 3:**
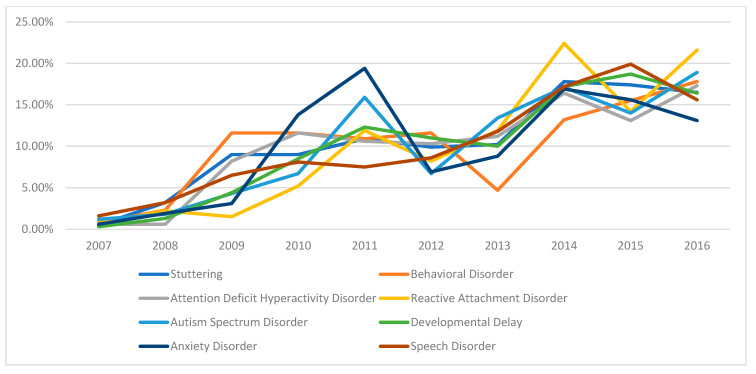
Distribution of diagnoses by year.

In this retrospective review spanning ten years of child psychiatry outpatient visits, the frequency of receiving a psychiatric diagnosis was higher during the first five-year period compared to the second five-year period. Annual admission rates and diagnostic rates are presented in Figure 1, while Figure 2 and Figure 3 illustrate the most common complaints and diagnoses, respectively. These figures demonstrate a yearly increase in referrals to the child psychiatry clinic.

Patient data were categorized based on presenting complaints and final diagnoses. The most frequent complaints were “delay in speech development”, “irritability/frustration”, “hyperactivity”, “request for a medical report”, and “stuttering”. Upon grouping these complaints into diagnosed and undiagnosed categories, it was observed that complaints of “delayed speech development” (*p* < 0.001), “stuttering” (*p* < 0.001), and “lack of eye contact” (*p* < 0.001) were significantly more common among patients who received a psychiatric diagnosis. Except for these three complaints, statistically significant differences were found between diagnosed and undiagnosed groups for other complaints. Detailed comparisons are presented in Table 2.

An analysis of diagnostic status revealed significant sex differences, with boys receiving diagnoses more frequently than girls (*p* < 0.001). Disorders such as Attention Deficit Hyperactivity Disorder (ADHD) (*p* < 0.001), Autism Spectrum Disorder (ASD) (*p* = 0.028), RAD (*p* < 0.001), Behavioral Disorder (*p* = 0.017), and tic disorder (*p* = 0.014) were more prevalent in boys. In contrast, anxiety disorder (*p* < 0.001), intellectual disability (*p* = 0.014), childhood masturbation (*p* < 0.001), and sleep disorder (*p* = 0.023) were more common in girls. Additionally, boys tended to be older at the time of admission (*p* = 0.007) and had longer clinical follow-up periods (*p* < 0.001) (Table 3).

**Figure 4:**
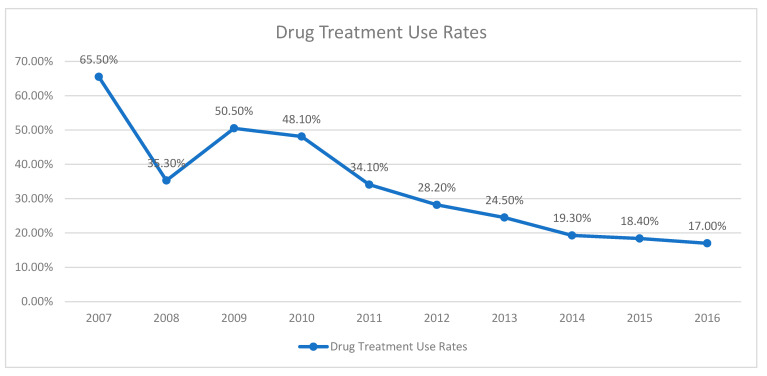
Change in drug use rates over the years.

The initiation of psychotropic medication, commonly prescribed medications, and associated side effects were also evaluated. Medication was initiated in 26.9% of cases, with risperidone (55.3%), hydroxyzine (23.8%), and methylphenidate (12.0%) being the most frequently prescribed. Side effect data were inconsistently documented; however, the available data indicated that the most common side effects were loss of appetite (35.3%) and drowsiness (20.6%) (Table 4). The initiation of psychotropic medications appears to vary over the years (Figure 4).

Comparisons between diagnosed and undiagnosed groups showed that diagnosed cases were older, had higher rates of medication initiation (including medication initiation during the first visit), and longer follow-up durations. Risperidone was predominantly prescribed in diagnosed patients, while hydroxyzine was more commonly used in undiagnosed patients (Table 5).

A backward logistic regression analysis was performed to identify the presenting complaints most predictive of initiating medication treatment. The model incorporated several variables, including sex, age, parental education levels, parental psychiatric illness status, and a range of specific presenting complaints: stuttering, inability to speak, irritability/frustration, hyperactivity, impaired comprehension, nail biting, bedwetting, soiling, lack of eye contact, sibling jealousy, aggression, frequent crying, not responding to one’s name, school refusal, childhood masturbation, inattention, sleep problems, feeding problems, fears, difficulty separating from the mother, discomfort with one’s gender, obsessions, somatic complaints, tics, and stereotypical behaviors.

Increasing age significantly elevated the likelihood of medication initiation [Exp(B) = 1.026; *p* < 0.001]. Lower paternal education levels significantly influenced medication use in the overall model (*p* = 0.001), although the individual educational categories (primary, secondary, tertiary) were not independently significant. Furthermore, the presence of psychiatric illness in fathers increased medication initiation odds by approximately 1.7-fold [Exp(B) = 1.688; *p* = 0.023]. Complaints that significantly raised the likelihood of medication use included inability to speak [Exp(B) = 1.274; *p* = 0.038], irritability/frustration [Exp(B) = 1.576; *p* < 0.001], hyperactivity [Exp(B) = 3.408; *p* < 0.001], lack of eye contact [Exp(B) = 4.269; *p* < 0.001], aggression [Exp(B) = 1.526; *p* = 0.022], school refusal [Exp(B) = 1.811; *p* = 0.044], sleep problems [Exp(B) = 2.903; *p* < 0.001], and fears [Exp(B) = 1.906; *p* = 0.017] (Table 6).

A logistic regression analysis using the enter method was conducted to evaluate the influence of psychiatric diagnoses on medication initiation. All psychiatric diagnoses were included in the model, along with age, sex, and parental psychiatric illness history. Factors significantly associated with increased medication use included age, paternal psychiatric illness history, intellectual disability, conduct disorder, ADHD, RAD, ASD, sleep disorder, anxiety disorder, speech disorder, adjustment disorder, and depression (all *p* < 0.001). Conduct disorder (OR = 22.002) had the greatest effect on increasing medication use, followed by sleep disorder (OR = 17.603), ADHD (OR = 16.288), and anxiety disorder (OR = 11.253). Notably, speech disorder was associated with the reduced likelihood of medication initiation (OR = 0.476). Sex and maternal or paternal educational status did not significantly impact medication use in the regression analysis (Table 7).

## 4. Discussion

This study examined the 10 years following the establishment of the child psychiatry unit and the initiation of outpatient services at our institution. During this period, detailed evaluations were conducted on admissions of children aged 0–6 years, including presenting complaints, diagnoses, treatments, side effects, and factors associated with initiating medication.

We assessed the annual distribution of admissions and diagnostic status of patients over the decade. The number of outpatient admissions increased progressively each year. The initially low admission rates during the first two years can be attributed to the unit being newly established and limited by the small number of available doctors, restricting appointment availability. Nevertheless, admissions steadily rose in subsequent years. Notably, while the diagnosis rate was higher during the first five years relative to the number of admissions, it declined during the latter five years. The gradual increase in outpatient admissions may reflect growing societal awareness about mental health, heightened parental focus on children’s education and development, and improved accessibility to health services. Furthermore, increased consultations without significant complaints suggest a reduced stigma associated with psychiatric evaluations, indicating that societal fears of psychiatric labeling might be diminishing. However, this trend may be specific to the region in which the study was conducted, as demographic, cultural, and socioeconomic factors can influence help-seeking behaviors; therefore, further studies in different settings would be valuable to determine the generalizability of these observations.

In this study, 64.6% of the patients were boys, possibly reflecting the greater prominence and higher prevalence of neurodevelopmental disorders in boys during early childhood, contributing to their increased referral rates. The predominance of boys may also be related to their slower physiological maturation compared to girls [21]. This pattern aligns with the findings from Muzi Li et al. (2019), which showed higher male admission rates (60.8%) in children aged 6–11 years but higher female admission rates (56.6%) in adolescents aged 12–17 years [22]. Similar male predominance has been consistently reported in other studies examining child psychiatric outpatient admissions across different years and countries [10,19,23,24,25].

Regarding diagnostic rates, 56.5% of cases received a psychiatric diagnosis in our study. Diagnosis rates in child psychiatry clinics vary widely in the literature [6,14]. For example, Karabekiroğlu et al. reported psychiatric or developmental problems in 15–45% of children aged 1–4 years [19]. A community-based study found that 41.7% of 204 children aged 3–6 years had at least one mental disorder [20]. Another study utilizing the Affective Disorders and Schizophrenia Interview Schedule for School-Age Children—Present and Lifetime Version reported at least one psychiatric diagnosis in over half (65%) of children aged 17–72 months [21].

When presenting complaints were analyzed in relation to psychiatric diagnoses, it was observed that children who did not make eye contact or did not respond to their name were more frequently diagnosed. This association is likely due to these symptoms being essential indicators for diagnosing ASD, a common diagnosis within this age group. Additionally, cases presenting with speech delays, comprehension difficulties, and GDD were also significantly associated with psychiatric diagnoses. These symptoms may indicate GDD as well as other neurodevelopmental disorders. Given that GDD is the most frequently diagnosed condition in the 0–6 age group [26], the early recognition of these symptoms, public awareness initiatives, and timely evaluations by child psychiatrists are essential. Other complaints significantly associated with receiving a diagnosis included stuttering, urinary incontinence, soiling, masturbation, and tics. The higher diagnostic rate for these specific symptoms is expected, given their strong association with specific psychiatric conditions. Some patient visits involved requests solely for medical reports, including educational support, financial aid, rehabilitation, or renewing expired reports. Because such reports require documented medical conditions as per the Regulation on the Issue of Medical Board Reports, these patients often fell into the diagnosed category.

The analysis revealed that specific complaints were significantly associated with psychiatric diagnoses, while others were not. Complaints with lower diagnostic rates included counseling requests without specific concerns, toileting problems, anxiety, sibling jealousy, nail biting, frequent crying, physical complaints, and gender dissatisfaction. The considerable number of families seeking consultation without significant symptoms, along with the rising referrals for developmental concerns, reflects increasing parental awareness regarding child psychiatry diagnoses and symptoms, which is an encouraging development.

Irritability/frustration was commonly reported upon admission to child psychiatric outpatient clinics [10,18]. In our study, irritability/frustration was associated with the absence of a psychiatric diagnosis. While studies in older age groups have shown higher diagnostic rates for this complaint [27], irritability in the 0–6 age group might often be perceived as a standard developmental characteristic.

When comparing the frequency of psychiatric diagnoses between sexes, our study found a higher diagnosis rate among boys. Specifically, ADHD, ASD, RAD, Behavioral Disorders, and tic disorders were more common in boys, whereas anxiety disorders, sleep disorders, and childhood masturbation were more frequent in girls. Neurodevelopmental disorders are generally more prevalent in boys, a finding consistent with other studies involving similar and different age groups [10,18,27,28]. Another study in a comparable age group also reported higher rates of anxiety disorders and childhood masturbation among girls [18]. Sleep problems, which can also point to underlying psychiatric conditions, vary in prevalence and present differently between genders according to the literature. An additional notable finding from our study was a higher incidence of intellectual disability among girls, contrasting with typical findings in the literature. This discrepancy might be due to younger children often initially receiving a diagnosis of GDD, with differentiation from intellectual disability occurring later.

Another important observation was that boys were older at the time of their initial presentation and had longer durations of clinical follow-up. The extended follow-up in boys may relate to their higher diagnostic rates. Older age at first presentation suggests that families might have initially anticipated symptom improvement with age, seeking help only as symptoms intensified. Supporting this hypothesis, patients in the diagnosed group were generally older, had longer clinical follow-up, and were more likely to initiate medication during their first appointment.

Our study also investigated psychotropic medication use in children aged 0–6 years. Psychotropic medication use has been rising in recent years, though data regarding changes in medication prescribing trends within a single center over time in this age group remain limited. Despite increased outpatient admissions to our clinic over the past 10 years (Figure 1, Figure 2 and Figure 3), we observed a decrease in the initiation rates of psychotropic medications (Figure 4). The rising number of referrals might reflect greater public awareness and sensitivity towards child psychiatric concerns, leading to more consultations involving milder symptoms or preventive advice rather than severe psychiatric presentations requiring medication. Emphasizing family involvement and appropriate psychoeducational programs or non-pharmacological interventions may have effectively reduced symptom severity, further decreasing the necessity for medication. However, low rates of regular follow-up could indicate that some patients recommended for medication may have discontinued their care prematurely, contributing to the overall low rate of medication initiation.

Risperidone, fluoxetine, hydroxyzine, and methylphenidate are among the most commonly prescribed medications for children aged 0–6 years [14,19]. One study evaluating medication use in children of this age compared those aged 1–3 with those aged 4–5 and found that medication use was substantially higher among the 4–5 age group [19]. In our own study, the most frequently prescribed medications were risperidone, hydroxyzine, methylphenidate, and fluoxetine.

Notably, drug treatments were also prescribed for some children who did not have a psychiatric diagnosis, with hydroxyzine used significantly more often in this undiagnosed group. This may be because hydroxyzine exerts a moderate sedative and anxiolytic effect, is helpful for sleep disorders, and generally has relatively few reported side effects [29]. Similar findings in other studies show that hydroxyzine is often preferred in the 0–6 age group [19,25,30]. Its higher rate of use in undiagnosed children in our study may be linked to subthreshold symptoms of anxiety and sleep disturbances that did not fulfill the criteria for a formal psychiatric diagnosis, making hydroxyzine an appealing treatment option.

A further aim of our study was to investigate the factors predicting the initiation of drug treatment in children aged 0–6. We found that older age, paternal psychiatric illness, and presenting complaints such as delayed speech, irritability/frustration/aggression, hyperactivity, poor eye contact, school refusal, sleep problems, and anxiety were associated with an increased likelihood of starting medication. In terms of diagnoses, intellectual disability, Behavioral Disorder, ADHD, RAD, ASD, sleep disorders, and anxiety disorders were positively associated with initiating treatment, whereas speech disorders showed a negative association.

These results align with previous research. In a study of infants and preschoolers (0–5 years), Uygun et al. reported a considerably higher rate of medication initiation among children aged 4–5 years compared to those aged 2–3 years [19]. Fontanella et al. similarly noted that being 4–5 years old was linked to greater medication use in preschool children between 2002 and 2008 [31]. Another study examining psychotropic drug use in young children (0–5 years) from 1994 to 2009 also found higher rates among boys and children aged 4–5 [32]. Consistent with these findings, our data show that medication use rises with age. We additionally observed that older children at the time of presentation were more likely to receive a psychiatric diagnosis—possibly because families become increasingly concerned about developmental problems as children grow older—and a higher number of formal diagnoses may, in turn, lead to the more frequent initiation of pharmacological treatment.

When evaluating the psychiatric status of parents, we found that a father’s psychiatric illness was associated with an increased likelihood of medication use in the child. This may indicate a significant genetic contribution, particularly since neurodevelopmental disorders are more frequently observed in males. However, it is important to interpret these findings cautiously, as the information on parental psychiatric conditions was based solely on family reports, and no formal psychiatric evaluations were conducted for the parents.

When examining specific diagnoses, we observed that intellectual disability, BD, ADHD, RAD, ASD, sleep disorders, and anxiety disorders predicted higher medication use. In the study by Uygun et al., anxiety, ADHD, and pervasive developmental disorder were identified as predictors of drug use [19]. Similarly, Fontanella et al. found that being 4–5 years old and male, as well as having ADHD, bipolar disorder, or disruptive behavior disorder, was associated with greater medication use in preschool children [31]. Consistent with these findings, our study and other evaluations of children aged 0–6 suggest that pharmacological treatment is frequently utilized. Although behavioral strategies and family-centered interventions can help manage psychiatric symptoms, medication may still be necessary in many cases.

In contrast to other psychiatric diagnoses, we found a negative correlation between speech and language disorders and medication use. Children with these disorders are typically referred for speech and language therapy, and medication is rarely prescribed unless there is a comorbid condition. As children’s communication skills improve with therapy, secondary behavioral symptoms—such as anger, irritability, or frequent crying—stemming from frustration at being unable to express themselves may also decrease.

Another noteworthy finding is that the complaint of “lack of speak” and the formal diagnosis of a speech impairment had different effects on medication use. We found a positive correlation between the parental complaint of the child’s inability to speak and medication use, possibly because not every child presenting with delayed speech meets diagnostic criteria for a primary speech or language disorder. Delayed speech can also be a symptom of ASD, intellectual disability, or GDD.

## 5. Conclusions

Our study identifies key clinical factors associated with the initiation of psychotropic medications in children aged 0–6 years, offering important insights into early childhood psychiatric treatment practices. We demonstrate that pharmacological interventions are frequently employed for certain psychiatric diagnoses, although non-pharmacological approaches remain foundational. Notably, our findings also reveal that psychotropic medications—particularly agents such as hydroxyzine—may be prescribed even in the absence of a formal psychiatric diagnosis, highlighting off-label use patterns in this population.

Several limitations must be acknowledged. The retrospective single-center design, based on data from patients who presented to a tertiary academic medical center, restricts the generalizability of the results and may not fully represent the broader population of the region. Additionally, the 10-year study period encompassed the transition from the DSM-IV-TR to the DSM-5, introducing potential variability in diagnostic classification. The inherent difficulty of diagnosing psychiatric conditions in very young children also presents challenges to diagnostic precision. Nonetheless, a key strength of our study lies in its large sample size and the comprehensive review of real-world clinical data over a decade.

This research contributes to the limited body of literature on early childhood psychopharmacology by systematically detailing prescribing trends, diagnostic profiles, and associated clinical-decision-making factors within a large cohort of preschool-aged children. Our results underscore the need for the careful evaluation of pharmacological treatment indications in early childhood, particularly in cases where a clear diagnostic framework is lacking.

Given the developmental sensitivity of this age group, future research should prioritize multi-center, prospective, and longitudinal studies to assess the long-term outcomes and safety profiles of early psychotropic medication use. Studies focusing on children without formal diagnoses are especially needed to clarify risk–benefit ratios. Additionally, the development of standardized, age-appropriate, and evidence-based guidelines is crucial to help clinicians determine when to initiate pharmacotherapy versus recommending psychosocial or behavioral interventions.

Finally, future research should explore the integration of parental mental health screening into routine child psychiatric assessments, as family dynamics and caregiver well-being may significantly influence treatment decisions. Expanding this research area will not only strengthen clinical practice, but also contribute to the development of more holistic, individualized, and ethically grounded care models for young children.

## Figures and Tables

**Table 1:** Sociodemographic features of the study sample.

		Mean ± s.d./n (%)
Age (months)		46.59 ± 16.48 (Min 1-Max 72, Median 48)
Sex	Girl	1174 (35.4%)
	Boy	2138 (64.6%)
School/nursery status	Kindergarten	540 (16.3%)
	Nursery	372 (11.2%)
	Special education	116 (3.5%)
	Not going to school	1741 (52.6%)
	First grade	121 (3.7%)
	Missing	422 (12.7%)
Mother’s education	Illiterate	80 (2.4%)
	Primary–secondary school	1197 (36.1%)
	High school	770 (23.2%)
	University–college	560 (16.9%)
	Missing	713 (21.5%)
Mother’s psychiatric illness	Yes	298 (9.0%)
	No	2404 (72.6%)
	Missing	618 (18.7%)
Father’s education	Illiterate	40 (1.2%)
	Primary–secondary school	1095 (33.1%)
	High school	834 (25.2%)
	University–college	612 (18.5%)
	Missing	739 (22.3%)
Father’s psychiatric illness	Yes	102 (3.1%)
	No	2559 (77.3%)
	Missing	659 (19.9%)

**Table 2:** Comparison of diagnostic status based on presenting complaints.

Presenting Complaint	Diagnosed	Undiagnosed	*p*
	n (%)	n (%)	
Speech delay	531 (28.4%)	275 (19.1%)	**0.000 ^X2^**
Irritability	337 (18.0%)	412 (28.6%)	**0.000 ^X2^**
Hyperactivity	309 (16.5%)	253 (17.5%)	0.438 ^X2^
Application for a medical report	273 (14.6%)	84 (5.8%)	**0.000 ^X2^**
Stuttering	243 (13.0%)	37 (2.6%)	**0.000 ^X2^**
Aggression	102 (5.5%)	88 (6.1%)	0.426 ^X2^
Inattention	95 (5.1%)	61 (4.2%)	0.252 ^X2^
Lack of eye contact	81 (4.3%)	22 (1.5%)	**0.000 ^X2^**
Nail biting	63 (3.4%)	102 (7.1%)	**0.000 ^X2^**
Bedwetting	63 (3.4%)	24 (1.7%)	**0.002 ^X2^**
Soiling	62 (3.3%)	10 (0.7%)	**0.000 ^X2^**
Toilet training problems	25 (1.3%)	49 (3.4%)	**0.000 ^X2^**
Consultation	18 (1.0%)	122 (8.5%)	**0.000 ^X2^**
Sleep problems	45 (2.4%)	50 (3.5%)	0.070 ^X2^
Social withdrawal	45 (2.4%)	42 (2.9%)	0.366 ^X2^
Fears	34 (1.8%)	47 (3.3%)	**0.008 ^X2^**
Impaired comprehension	38 (2.0%)	12 (0.8%)	**0.005 ^X2^**
Developmental delay	46 (2.5%)	12 (0.8%)	**0.000 ^X2^**
Sibling jealousy	16 (0.9%)	50 (3.5%)	**0.000 ^X2^**
Frequent crying	30 (1.6%)	57 (4.0%)	**0.000 ^X2^**
Not responding to one’s name	54 (2.9%)	13 (0.9%)	**0.000 ^X2^**
School refusal	35 (1.9%)	36 (2.5%)	0.218 ^X2^
Masturbation	36 (1.9%)	7 (0.5%)	**0.000 ^X2^**
Eating issues/feeding problems	36 (1.9%)	39 (2.7%)	0.167 ^X2^
Difficulty separating from the mother	29 (1.6%)	18 (1.2%)	0.465 ^X2^
Easily bored	12 (0.6%)	12 (0.8%)	0.522 ^X2^
Discomfort with one’s gender	2 (0.1%)	10 (0.7%)	**0.005 ^X^** ^2^
Obsessions	24 (1.3%)	29 (2.0%)	0.098 ^X2^
Somatic complaints	6 (0.3%)	22 (1.5%)	**0.000 ^X2^**
Tics	22 (1.2%)	0 (0.0%)	**0.000 ^X2^**
Frequent lying	3 (0.2%)	2 (0.1%)	0.873 ^X2^
Stereotypical behaviors	24 (1.3%)	11 (0.8%)	0.146 ^X2^
Picky eating	0 (0.0%)	1 (0.1%)	0.435 ^X2^

X^2^: Chi-Square test. Note: Table based on Chi-Square test analysis data. Bold numbers indicate the statistically significant differences.

**Table 3:** Comparison of diagnoses by sex.

		Girls	Boys	*p*
		Mean ± s.d. (Median)/n (%)	Mean ± s.d. (Median)/n (%)	
Age (months)		45.5 ± 16.7 (46)	47.2 ± 16.3 (48)	**0.007 ^m^**
Diagnosis				**0.000 ^X2^**
Yes		614 (52.3%)	1256 (58.7%)	
No		560 (47.7%)	882 (41.3%)	
Missing		2	6	
Diagnosis count	1	549 (89.4%)	1118 (89.0%)	0.794 ^X2^
	2	60 (9.8%)	131 (10.4%)	
	3	4 (0.7%)	6 (0.5%)	
	4	1 (0.2%)	1 (0.1%)	
Developmental Delay		143 (23.3%)	247 (19.7%)	0.592 ^X2^
ADHD		61 (9.9%)	268 (21.3%)	**0.000 ^X2^**
Stuttering		83 (13.5%)	138 (11.0%)	0.497 ^X2^
Speech Disorder		59 (9.6%)	127 (10.1%)	0.274 ^X2^
ASD		45 (7.3%)	119 (9.5%)	**0.028 ^X2^**
Anxiety Disorder		81 (13.2%)	79 (6.3%)	**0.000 ^X2^**
RAD		26 (4.2%)	108 (8.6%)	**0.000 ^X2^**
Behavioral Disorder		33 (5.4%)	96 (7.6%)	**0.017 ^X2^**
Encopresis		18 (2.9%)	51 (4.1%)	0.100 ^X2^
Enuresis		27 (4.4%)	33 (2.6%)	0.118 ^X2^
Intellectual Disability		29 (4.7%)	28 (2.2%)	**0.014 ^X2^**
Specific Learning Disorder		11 (1.8%)	24 (1.9%)	0.617 ^X2^
Masturbation		24 (3.9%)	10 (0.8%)	**0.000 ^X2^**
Sleep Disorder		17 (2.8%)	14 (1.1%)	**0.023 ^X2^**
Tic Disorder		3 (0.5%)	22 (1.8%)	**0.014 ^X2^**
Eating Disorder		3 (0.5%)	9 (0.7%)	0.449 ^X2^
Adjustment Disorder		5 (0.8%)	7 (0.6%)	0.652 ^X2^
Obsessive Compulsive Disorder		4 (0.7%)	6 (0.5%)	0.763 ^X2^
Depression		5 (0.8%)	3 (0.2%)	0.109 ^X2^
Trichotillomania		3 (0.5%9)	2 (0.2%)	0.354 ^X2^
Neglect/Abuse		1 (0.2%)	2 (0.2%)	1.000 ^X2^
Stereotypic Movement Disorder		1 (0.2%)	2 (0.2%)	1.000 ^X2^
Gender Dysphoria		0 (0.0%)	1 (0.1%)	1.000 ^X2^
Somatization Disorder		1 (0.2%)	0 (0.0%)	0.354 ^X2^
Clinical follow-up duration				**0.002 ^X2^**
Single evaluation		573 (48.5%) ^a^	932 (43.5%) ^b^	
1–6 months		355 (30.1%) ^a^	637 (29.8%) ^a^	
7–12 months		105 (8.9%) ^a^	204 (9.5%) ^a^	
1–3 years		90 (7.6%) ^a^	189 (8.8%) ^a^	
4–5 years		39 (3.3%) ^a^	112 (5.2%) ^b^	
>5 years		19 (1.6%) ^a^	67 (3.1%) ^b^	

X^2^: Chi-Square test; m: Mann–Whitney U test. Note: Table based on Chi-Square test analysis data. Bold numbers indicate the statistically significant differences. Different letters (a,b) indicate the statistically significant group differences by Bonferroni correction. ADHD—Attention Deficit Hyperactivity Disorder, ASD—Autism Spectrum Disorder, RAD—Reactive Attachment Disorder.

**Table 4:** Most frequently used drug treatments and their side effects.

		Mean ± s.d./n (%)
Medication use		
Yes		894 (26.9%)
No		2410 (72.6%)
Missing		15 (0.5%)
Medication prescribed at the first visit	Yes	419 (12.6%)
	No	2886 (86.9%)
	Missing	15 (0.5%)
Medication initiation session		2.82 ± 2.189 (Min 1–Max 20, Median 2)
Medication		
Risperidone		494 (55.3%)
Hydroxyzine		213 (23.8%)
Methylphenidate		107 (12.0%)
Fluoxetine		59 (6.6%)
Atomoxetine		16 (1.8%)
Ecsitalopram		6 (0.7%)
Alprazolam		1 (0.1%)
Aripiprazole		2 (0.2%)
Melatonin		2 (0.2%)
Piracetam		2 (0.2%)
İmipramine		1 (0.1%)
Medication-related side effect, Yes		34 (3.8%)
Loss of appetite		12 (35.3%)
Drowsiness		7 (20.6%)
Insomnia		4 (11.8%)
Irritability		3 (8.8%)
Lethargy		2 (5.9%)
Nausea		2 (5.9%)
Enuresis		1 (2.9%)
Headache		1 (2.9%)
Rash		1 (2.9%)
Dark circles under the eyes		1 (2.9%)
Weight gain		1 (2.9%)
Hallucination		1 (2.9%)

**Table 5:** Comparison of medical treatments and side effects in diagnosed and undiagnosed groups.

		Diagnosed	Undiagnosed	*p*
		Mean ± s.d. (Median)/n (%)	Mean ± s.d. (Median)/n (%)	
Month of application		47.6 ± 16.9 (48)	45.3 ± 15.9 (45)	**0.000 ^m^**
Clinical follow-up duration		2.41 ± 1.466 (2)	1.58 ± 0.861 (1)	**0.000 ^m^**
At which consultation medication was prescribed		2.9 ± 2.2 (2)	2.6 ± 2.1 (2)	0.354 ^m^
Medication prescribed at the first visit				**0.000 ^X2^**
Yes		341 (18.3%)	77 (5.4%)	
No		1522 (81.7%)	1359 (94.6%)	
Missing		7	6	
Medication usage	Yes	741 39.8	152 10.6	**0.000 ^X2^**
	No	1122 60.2	1283 89.3	
	Missing	7	7	
Medication				
Risperidone		422 (57%)	71 (46.7%)	**0.021 ^X2^**
Hydroxyzine		145 (19.6%)	68 (44.7%)	**0.000 ^X2^**
Methylphenidate		102 (13.8%)	5 (3.3%)	**0.000 ^X2^**
Fluoxetine		54 (7.3%)	5 (3.3%)	0.071 ^X2^
Atomoxetine		14 (1.9%)	2 (1.3%)	0.627 ^X2^
Ecsitalopram		5 (0.7%)	1 (0.7%)	1.000 ^X2^
Alprazolam		1 (0.1%)	0	1.000 ^X2^
Aripiprazole		2 (0.3%)	0	1.000 ^X2^
Melatonin		2 (0.3%)	0	1.000 ^X2^
Piracetam		2 (0.3%)	0	1.000 ^X2^
İmipramine		1 (0.1%)	0	1.000 ^X2^
Medication-related side effect, Yes		28 (3.8%)	6 (4%)	0.895 ^X2^
Drowsiness		5 (17.9%)	2 (33.3%)	0.334 ^X2^
Loss of appetite		11 (39.3%)	1 (16.7%)	0.429 ^X2^
Nausea		1 (3.6%)	1 (16.7%)	0.308 ^X2^
Enuresis		1 (3.6%)	2 (33.3%)	0.075 ^X2^
Headache		1 (3.6%)	0	1.000 ^X2^
Rash		1 (3.6%)	0	1.000 ^X2^
Insomnia		4 (14.3%)	0	1.000 ^X2^
Dark circles under the eyes		1 (3.6%)	0	1.000 ^X2^
Irritability		3 (10.7%)	0	1.000 ^X2^
Weight gain		1 (3.6%)	0	1.000 ^X2^
Stagnation		2 (7.1%)	0	1.000 ^X2^
Hallucination		1 (3.6%)	0	1.000 ^X2^

X^2^: Chi-Square test; m: Mann–Whitney U test. Different letters indicate the statistically significant group differences by Bonferroni corretion. Note: Table based on Chi-Square test analysis data. Bold numbers indicate the statistically significant differences. m: Mann–Whitney U test.

**Table 6:** Logistic regression analysis of factors associated with drug treatment initiation based on presenting complaints.

Variables	B	S.E.	Sig.	Exp(B)	95% C.I. for EXP(B)	
					Lower	Upper
Age (month)	0.026	0.003	**0.000**	1.026	1.020	1.033
Father’s education						
Illiterate			**0.001**			
Primary–secondary school	−0.083	0.392	0.832	0.920	0.427	1.983
High school	−0.307	0.395	0.438	0.736	0.339	1.596
University–college	−0.592	0.401	0.140	0.553	0.252	1.214
Father’s mental illness (Yes)	0.523	0.231	**0.023**	1.688	1.074	2.652
Presenting complaints						
Speech delay	0.242	0.117	**0.038**	1.274	1.014	1.602
Irritability	0.455	0.111	**0.000**	1.576	1.269	1.959
Hyperactivity	1.226	0.116	**0.000**	3.408	2.717	4.275
Lack of eye contact	1.451	0.256	**0.000**	4.269	2.585	7.051
Aggression	0.423	0.185	**0.022**	1.526	1.062	2.194
School refusal	0.594	0.295	**0.044**	1.811	1.015	3.230
Sleep problems	1.066	0.253	**0.000**	2.903	1.767	4.770
Fears	0.645	0.270	**0.017**	1.906	1.123	3.237
Difficulty separating from mother	0.649	0.366	0.076	1.914	0.934	3.923
Constant	0.770	0.405	0.057	2.159		

Note: This table is based on logistic regression analysis using the stepwise LR method. Bold numbers indicate the statistically significant differences.

**Table 7:** Logistic regression analysis of diagnostic predictors of drug treatment initiation.

Variables	B	S.E.	Sig.	Exp(B)	95% C.I. for EXP(B)	
					Lower	Upper
Sex (Male)	0.157	0.119	0.189	1.170	0.926	1.477
Age (month)	0.011	0.004	**0.002**	1.012	1.004	1.019
Mother’s education						
Illiterate			0.650			
Primary–secondary school	0.198	0.354	0.575	1.220	0.609	2.441
High school	0.177	0.370	0.633	1.193	0.578	2.462
University–college	−0.007	0.391	0.986	0.993	0.462	2.136
Mother’s mental illness (Yes)	−0.077	0.183	0.673	0.926	0.647	1.324
Father’s education						
Illiterate			0.406			
Primary–secondary school	0.160	0.473	0.736	1.173	0.464	
High school	0.057	0.485	0.907	1.059	0.409	
University–college	−0.139	0.500	0.780	0.870	0.327	
Father’s mental illness (Yes)	0.684	0.267	**0.010**	1.982	1.175	3.344
Diagnoses						
Stuttering	0.138	0.233	0.555	1.147	0.726	
Mental retardation	0.585	0.174	**0.001**	1.795	1.276	2.526
Behavioral disorder	3.091	0.287	**0.000**	22.002	12.529	38.636
ADHD	2.790	0.176	**0.000**	16.288	11.536	22.999
Reactive attachment disorder	1.453	0.235	**0.000**	4.274	2.697	
Externalizing disorder	−0.090	0.314	0.775	0.914	0.494	
ASD	2.269	0.205	**0.000**	9.671	6.472	14.451
Sleep disorder	2.868	0.447	**0.000**	17.603	7.327	42.292
Anxiety disorders	2.421	0.203	**0.000**	11.253	7.556	
Speech disorder	−0.742	0.303	**0.014**	0.476	0.263	0.862
Childhood masturbation	−0.970	1.027	0.345	0.379	0.051	2.834
Adjustment disorder and depression	1.530	0.573	**0.008**	4.619	1.502	14.205
Tic disorder	0.289	0.617	0.640	1.334	0.398	
Constant	5.641	1.026	**0.000**	281.688		

Note: Table based on logistic regression analysis using the enter method. Bold numbers indicate the statistically significant differences. ADHD—Attention Deficit Hyperactivity Disorder.

## Data Availability

The data presented in this study are available on request from the corresponding author due to privacy restrictions.
